# Long-Term Diet Quality and Risk of Diabetes in a National Survey of Chinese Adults

**DOI:** 10.3390/nu14224841

**Published:** 2022-11-16

**Authors:** Yumeng Hua, Ziwei Zhang, Aiping Liu

**Affiliations:** Department of Social Medicine and Health Education, School of Public Health, Peking University Health Science Center, Beijing 100191, China

**Keywords:** diet quality, diabetes, diet balance index, China health and nutrition survey, CHNS

## Abstract

There is little evidence involving the association between diet quality and the risk of diabetes among the Asian populations, especially from the long-term prospective cohort studies in China. This study evaluated the long-term diet quality of Chinese adults by the Chinese diet balance index 2016 (DBI-16) and firstly explored its role in diabetes prevention. A total of 9394 participants from the China health and nutrition survey (2004–2015) prospective cohort were included. Dietary information was selected by three consecutive 24-h dietary recalls, combined with a household food inventory and further calculated as the scores of the DBI-16 components and indicators. Three major indicators, the low bound score (LBS), the high bound score (HBS) and the diet quality distance (DQD), were divided into four level groups, according to the total scores, respectively, including Level 1 (scores below 20%), Level 2 (20–40% of scores), Level 3 (40–60% of scores) and Level 4 (scores above 60%). Diabetes cases were identified through a questionnaire or by testing the overnight fasting blood samples. Cox’s proportional hazards models were used to estimate the hazard ratios (HRs) and 95% CIs, while restricted cubic splines (RCS) were applied to explore the potentially non-linear relationships. During a median follow-up of 6.0 years (61,979 persons-years), 657 participants developed diabetes. The LBS and DQD scores were positively associated with diabetes risks, whereas no significant association of the HBS scores with diabetes risks was observed. Compared with those on the lowest level, the adjusted HRs (95%) across the increased levels of diet quality were 2.43 (1.36, 4.37), 3.05 (1.69, 5.53) and 4.90 (2.46, 9.78) for the LBS; 1.06 (0.74, 1.51), 1.30 (0.99, 1.88) and 0.99 (0.39, 2.55) for the HBS; 1.28 (1.01, 1.61) and 2.10 (1.57, 2.82) for the DQD after pooling the participants on Level 1 and 2 as the reference group, due to the few who developed diabetics on Level 1 of the DQD. No significantly non-linear shape was observed for all three indicators. Our findings indicated a significant inverse association between the long-term diet quality assessed by the DBI-16 and diabetes risks, providing evidence for the positive role of healthy diets in diabetes prevention in Asia.

## 1. Introduction

Diabetes mellitus is a global health problem with a notable growth in both incidences and prevalence, contributing to huge health and economic burdens. Around 537 million adults, aged 20–79, were living with this disorder, which is predicted to rise to 643 million by 2030; the diabetes-related health expenditure was estimated at USD 966 billion dollars at least, which has increased by 316% over the last 15 years [1]. Over 75% of people with diabetes live in low- and middle-income countries [1], of which China is the country currently accounting for the largest proportion [2]. With rapid economic growth, China has seen an increasing prevalence of diabetes from 0.67% in 1980, to 11.2% in 2015–2017, with around 129.8 million patients affected on the mainland in 2017 [2,3,4]. Therefore, the prevention and management of diabetes have become an undoubted priority in the public health interest worldwide, especially in China.

A large body of studies have recognized the beneficial impact of healthy diets on diabetes prevention [5,6,7]. Considering people’s eating habits of consuming a combination of several foods and beverages, rather than single food groups, dietary indexes have emerged as a complementary tool to evaluate the overall diet quality of individuals and avoid the shortage of evaluating nutrients and foods in isolation, which are hypothesis-derived, based on scientific dietary recommendations [8]. Several majorly common indexes have been constructed, with reference to dietary guidelines of American or European countries or typical healthy dietary patterns among western populations, including the alternate Mediterranean diet (aMED), the dietary approaches to stop hypertension (DASH) diet score, the healthy eating index (HEI) and the alternate healthy eating index (AHEI), which have been reported, in association with a 13–21% reduced risks of diabetes [6,9,10].

Despite the preventive role of a higher quality diet on the development of diabetes, in previous analyses, it could not be ignored that most relative studies have been conducted among American and European adults, who have divergent characteristics of dietary habits, as well as genetic and metabolic backgrounds from Chinese adults, who use dietary indexes which are more appropriate to western populations. The Chinese diet balance index (DBI), with reference to the development of typical dietary indexes, including the HEI and the diet quality index (DQI), has been constructed, based on the Chinese dietary guidelines (CDG) and the food guide pagoda (FGP) [11]. Compared with other indexes, the DBI tends to center on the main dietary features of Chinese residents and has been a valuable tool to evaluate the associations of diet quality with health outcomes in China [12,13]. Whereas, there is less evidence using dietary indexes to explore the potential benefits of healthy diets on diabetes prevention among Asian people, especially among the Chinese population, whose diet quality was assessed by the DBI. Thus, for the first time, this study conducted a prospective analysis to examine the association between diet quality evaluated by the DBI and the risk of diabetes in a large population-based cohort of Chinese adults.

## 2. Materials and Methods

### 2.1. Study Design and Participants

The CHNS is an ongoing, large-scale, open and longitudinal cohort study in China, which was conducted in 10 rounds from 1989 to 2015. As a national health and nutrition survey, the CHNS has collected the data using a multistage, random cluster method from 15 provinces of China at the individual and household levels so far. The study was approved by the institutional review committees of the University of North Carolina and the National Institute of Nutrition and Food Safety from the Chinese Center for Disease Control and Prevention. All participants provided written informed consent. Details on the CHNS has been published previously [14].

Our analyses used five rounds of the CHNS data, from 2004 to 2015. Among the 34,012 participants from these rounds, we excluded individuals aged < 18 years at the baseline (*N* = 14313) and individuals who were pregnant, nursing and disabled (*N* = 934). We also excluded people with incomplete diabetes information (*N* = 3780), were lost to follow-up after the baseline or were first entered into the CHNS in 2015 (*N* = 4321), those without complete dietary data in the round before the end of the study (*N* = 737), those with missing or implausible energy intake information (>5000 or <700 kcal/day) (*N* = 53), and those with a history of diabetes, stroke, myocardial infarction or any type of tumor at the baseline (*N* = 480) (Supplementary Figure S1). Finally, a total of 9394 participants were included in this study.

### 2.2. Dietary Data Collection

In each survey round, the dietary data in the CHNS were collected by three consecutive 24-h dietary recalls at the individual level. Over the same three days, a weighing inventory was also conducted to weigh and record information on the edible oils and condiments consumption at the household level. The consumption of all foods for each round was collected by trained nutritionists, through a face-to-face interview, and categorized into groups, according to the China food composition tables (FCTs) and the introduction about the DBI-16 [15,16,17]. The United States Department of Agriculture database was used to calculate the consumption of added sugars, due to the lack of data on them in the FCTs.

### 2.3. DBI-16

As one of the most common dietary indexes used in China, the DBI was recommended to evaluate the overall diet quality of the Chinese residents, which was first constructed in 2005 and has been updated twice. The DBI-16, the latest version, based on the CDG and FGP (2016), could discover more accurately whether a person’s current dietary intake is varied and well-balanced.

The DBI-16 provides food recommendations for each of the 11 energy levels. It comprises seven components (involving 12 major food groups) with different scores ranging from −12 to 12, including: (1)cereal; (2) vegetable and fruit; (3) dairy, soybean and products, (4) animal food (further classified into meat, fish and egg); (5) empty energy food (further classified into cooking oils and alcoholic beverages); (6) condiments (further classified into addible sugar and salt); and (7) diet variety, involving 12 food groups. A score of 0 for each component indicates that the food consumption meets the lowest recommended amounts from the dietary guideline. A higher absolute value of scores indicates a more unbalanced food intake. The positive or negative scores signify that food intakes are excessive or insufficient, respectively.

The crucial indicators of the DBI-16 include the LBS, HBS and DQD, which are calculated, based on the scores of all aforementioned components. The LBS refers to the sum of the absolute values of all negative scores, representing insufficient food consumption with a 0–60 score range. The HBS refers to the sum of all positive scores, representing an excessive food consumption with a 0–44 score range. The DQD refers to the sum of the absolute value of both positive and negative scores, which evaluates whether the dietary habits are healthy and well-balanced, comprehensively.

These three indicators are further divided into four level groups, according to the total scores, including ‘almost no problem’ (Level 1, scores below 20% for the LBS: 0–12, HBS: 13–24, and DQD: 0–17), ‘low level of problem’ (Level 2, 20~40% of scores for the LBS: 13–24, HBS: 10–18, and DQD: 18–34), ‘moderate level of problem’ (Level 3, 40~60% of scores for the LBS: 25–36, HBS: 19–27, and DQD: 35–50) and ‘high level of problem’ (Level 4, scores above 60% for LBS: >36, HBS: >27, and DQD: >50). More detailed designs and standards of the DBI-16 were described elsewhere [15,18].

### 2.4. Definition of Diabetes

As the main outcome, diabetes was identified by the questionnaire inquiring in each round of surveys, since 2004. Three questions were involved in these questionnaires to collect individual information on the history of diabetes, including: (1) Has a doctor ever told you that you suffer from diabetes? (2) How old were you when the doctor told you this? and (3) Did you use any of these treatment methods of diabetes, for instance, special diet, weight control, oral medicine, injection of insulin, Chinese traditional medicine, home remedies and Qi Gong (spiritual method)? A self-reported diabetes diagnosis was considered if at least one of the answers to the aforementioned questions was “yes” for each survey. Moreover, overnight fasting blood samples were collected and assayed with a strict quality control in the 2009 round, so individuals with a fasting plasma glucose ≥7.0 mmol/L or HbA1c ≥ 6.5%, were also confirmed as diabetics in 2009. The year of the first diabetes diagnosis is defined as the middle year between the round of the first diagnosis and the nearest round before.

### 2.5. Other Variables

Data on the demographic and lifestyle behaviors were obtained through questionnaires at each round, including age, sex, residence, education level, occupation, smoking and drinking status, dietary intake (including the amounts of total energy, fat, carbohydrates and protein intake) and a history of hypertension. Body height and weight were measured with standard measuring procedures and calibrated equipment, which were further calculated for the body mass index (BMI) as the weight (kg) divided by the height squared (m^2^). Physical activity was calculated, based on individual occupational, transportation, domestic and leisure activities, from the questionnaire information, further divided into 3 levels (including inadequate (<600 MET-min/w), low (600–3999 MET-min/w), and medium or high (≥4000 MET-min/w) level)). In addition, all non-dietary covariates were confirmed with data from the baseline year, whereas fat, carbohydrate and protein intakes were estimated as the energy-adjusted cumulative average intake from the baseline and follow-up, and the total energy intakes were estimated as the cumulative average intake.

### 2.6. Statistical Analysis

The diet quality was evaluated by seven components and three major indicators (LBS, HBS and DQD) of the DBI-16 and then categorized into 4 level groups, according to the different levels of the LBS, HBS and DQD, respectively. As only one diabetic on Level 1 of the DQD was observed during surveys, we pooled participants on Level 1 and Level 2 of the DQD into one group during the analyses, which represented individuals who had rather balanced diets. For better assessing the individual long-term diet quality and minimizing within-person variations, all dietary scores were calculated as the cumulative average scores by averaging the repeated measurements in each round. We calculated each individual’s person-years from the baseline which is the year of each person’s first entry into the survey to a first diabetes diagnosis, the last survey round before the participant’s departure from the survey, or the end of the latest survey (2015), whichever came first. Incidence rates of diabetes, expressed as per 1000 person-years, were calculated by dividing the number of diabetes cases by person-years of follow-up in each level.

To understand the effect of various food groups on the scores of the DBI-16 indicators, we calculated the contribution rates of each sub-component to the LBS, HBS and DQD, respectively. Furthermore, to compare the differences in the population characteristics on the different levels of the DBI indicators, we used χ^2^ tests for the categorical variables presenting as percentages and the Kruskal–Wallis test for the continuous variables presenting as median (interquartile range, IQR). We used Cox’s proportional hazards to calculate the hazard ratios (HRs) and 95% CIs to estimate the association of the DBI indicators with the diabetes risks, adjusting for age, sex, education level and urban or rural residence at the baseline in model 1, for the above covariates in model 1 and physical activity, smoking and alcoholic drinking in model 2, and for the above covariates in model 2 and body mass index (BMI) and hypertension in model 3. Restricted cubic splines (RCS) were applied to display the potentially non-linear relationship of the DBI indicators with developed diabetes in a more intuitive way with adjustments for the covariates in model 3. Meanwhile, we conducted two sensitivity analyses by including all individuals with missing values of entire diabetes information from the questionnaires, and excluding all participants without entire information on the covariates in model 3. A two-sided *p* values < 0.05 was considered to indicate the statistical significance. All analyses were performed with R version 4.1.0.

## 3. Results

### 3.1. Characteristics of the DBI-16 Sub-Components on the Contribution

A total of 9394 participants were included in the final analyses. Table 1 shows the contribution rates of the DBI-16 sub-components on the LBS, HBS and DQD. The top five sub-components to the LBS were dairy (21.37%), diet variety (20.74%), fruit (19.13%), fish (10.18%) and soybean (9.97%), all of which accounted for 81.39% of the contribution rates to the LBS scores. The top five sub-components to the HBS were cereal (48.48%), salt (18.97%), oil (16.37%), meat (11.01%) and egg (3.92%), all of which accounted for 98.75% of the contribution rates to the HBS scores. The top five sub-components to the DQD were cereal (19.4%), dairy (13.0%), diet variety (12.61%), fruit (11.64%) and salt (7.44%), all of which accounted for 64.9% of the contribution rates to the DQD scores.

### 3.2. Baseline Characteristics of the Study Participants

The baseline characteristics of the participants with the LBS, HBS and DQD, were illustrated in Table 2. The median age of participants was 46 (IQR: 35; 57). Individuals on higher levels of the LBS or DQD had a higher physical activity level, lower educational level, a BMI and prevalence of hypertension. They were more likely to be male, farmers or workers, and smokers, while less likely to have higher percentages of urban residence. They also tended to consume more carbohydrates and less fat and protein. Moreover, participants on the higher levels of the LBS were less likely to be drinkers, whereas people on the higher levels of the DQD were more likely to be drinkers. On the higher levels of the HBS, the participants tended to male, smokers and drinkers. They had a higher physical activity level and lower total energy intakes.

### 3.3. Associations of Diet Quality with the Risks of Diabetes

During a median follow-up of 6.0 years (IQR: 4.0–11.0 years, 61,979 person-years), 657 participants (10.60 cases/1000 person-years) developed new-onset diabetes. Among them, 343 were those with self-reported physician-diagnosed diabetes during the follow-up period and 469 were confirmed as diabetes cases, based on the assay of blood samples. A total of 320 participants were under the glucose-lowering treatment.

**Figure 1:**
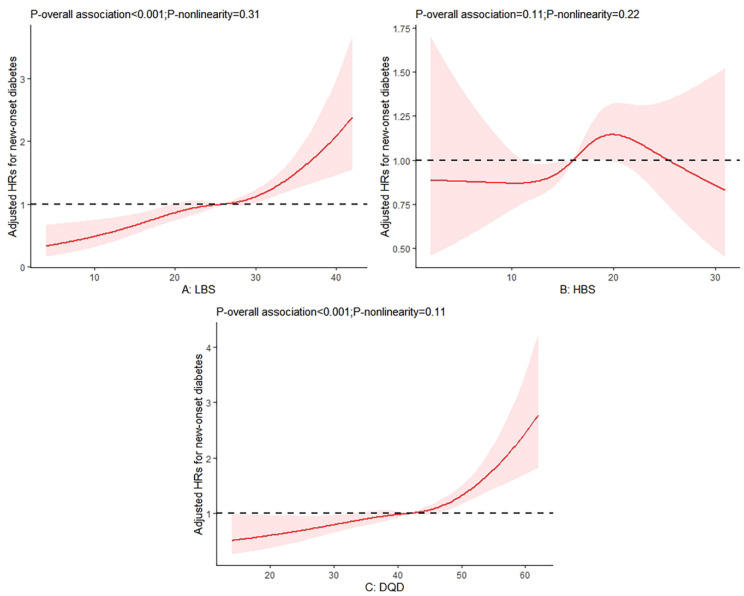
Relation of (**A**) the LBS, (**B**) HBS, and (**C**) DQD with the risks of diabetes. Note: Adjusted for age, sex, education level, urban or rural residence, physical activity, smoking, alcoholic drinking, body mass index (BMI), as well as hypertension.

Among the three major indicators, the LBS and DQD scores were positively associated with the risks of diabetes, whereas no significant association between the HBS scores and diabetes risks was observed (Table 3). Compared with those on Level 1 of the LBS, the adjusted HRs (95% CIs) in model 3 were 2.43 (1.36, 4.37) on Level 2, 3.05 (1.69, 5.53) on Level 3 and 4.90 (2.46, 9.78) on Level 4, respectively. For the HBS, the adjusted HR (95% CIs) across the extreme levels were 1.06 (0.74, 1.51) on Level 2, 1.30 (0.99, 1.88) on Level 3 and 0.99 (0.39, 2.55) on Level 4. Compared with those on Level 1 and 2 of the DQD, the adjusted HRs (95% CIs) in model 3 were 1.28 (1.01, 1.61) on Level 3 and 2.10 (1.57, 2.82) on Level 4. Moreover, no significantly non-linear relationship was observed for these three indicators (Figure 1).

Two sensitivity analyses were conducted in this study. First, as people without a diabetes history might not report related information in the actual survey, we included all participants with missing values of the complete diabetes information from the questionnaires into the analyses as individuals who do not have a diabetes diagnosis. We found this exclusion didn’t substantially change the results (Supplementary Table S1 and Figure S2). In addition, we examined the potential influence of filling in missing values of covariates, by excluding all participants without complete information on the covariates in model 3. The results from this analysis were similar to the main results (Supplementary Table S2 and Figure S3).

## 4. Discussion

In this large prospective cohort among Chinese adults, the three major indicators of the DBI-16 were calculated to evaluate the overall diet quality from different perspectives. A lower adherence to the DBI dietary patterns was associated with a higher risk of diabetes. Similar positive associations were observed for the LBS and DQD scores, without significant non-linear shapes, whereas the HBS scores had no statistically significant association with diabetes risks. Our findings fill the current knowledge gap in the association between healthy diets, based on the recommendations of the CDG and FGP and diabetes risks, and enrich the evidence about the dietary prevention of diabetes among Asian populations involving the Chinese.

Several healthy diets have been beneficial for diabetes prevention, including the Mediterranean, DASH, HEI and AHEI diets [19,20,21], all of which share common characteristics: high whole grain, vegetable, fruit, nut and legume intakes; moderate fish and dairy product intakes; and restricted refined grains, red and processed meat, sugar-sweetened beverages and added sodium intakes [22,23]. The DBI is developed with reference to some classical dietary indexes, such as the HEI and DQI, and contains the vast majority of the aforementioned dietary characteristics. Previous studies have supported the DBI as an effective tool to evaluate the diet quality of Chinese residents, in which a substantial change in diet has undergone. Several specific features of the dietary patterns in China are noteworthy: the population has experienced a dual burden of overnutrition and undernutrition; although the consumption of fats and animal-based foods keeps increasing year by year with the decreasing consumption of cereals, tubers and vegetables, cereals remain the main source of dietary energy; eggs, fish and dairy product intakes remain deficient, while edible oils, salt and added sugar intakes have exceeded recommendations [24,25,26]. As the DBI tends to center on the main dietary problems of Chinese residences, compared with other typical dietary indexes, it is more suitable to evaluate the diet quality in China and explore its association with diabetes risks.

As the main indicator of the DBI, the LBS contains nine food groups, among which dairy, diet variety, fruits, fish and soybean had the most contribution rates of the LBS scores in this study. Our findings highlight the significantly positive association between the LBS scores and diabetes risks, emphasizing the importance of considering the deficiency of these food intakes. Most previous prospective studies have supported dairy products, especially low-fat dairy products and yoghurt, to be the strong predictor of diabetes, including the evidence from a dose-response meta-analysis involving 22 cohort studies [27]. Despite inconsistent findings on the importance of the diet variety for diabetes prevention, a recent meta-analysis reported that a greater diet variety across the major food groups and with fruits and/or vegetables, had an inverse association with the risk of diabetes [28]. Evidence about the association of fruits and fish with diabetes risks remain controversial. A recent umbrella review summarized relevant systematic reviews, and did not find a significant benefit of high fruit and vegetable intakes and high fish intake in reducing the diabetes risk. When considering fruit separately, one systematic review reported the benefit of high intakes for diabetes prevention, whereas two systematic reviews reported no significant association [29]. Several systematic reviews about soybean showed relatively consistent results that soybean, soy products and soy constituents (including soy protein and soy isoflavones) might lower the diabetes risk with a higher intake [30,31,32]. Considering the possible protective effects of these food groups, their lack of intake might contribute to an increased diabetes risk.

Our study did not observe a significant association between the HBS scores and the diabetes risks, which could be attributed to several food groups accounting for the highest contribution rates of the HBS scores. Cereals, as the staple of Chinese residents provided half the contribution of the HBS scores in this study, implying that a large cereal intake has become the major cause of dietary excess. Previous studies reported a shift towards refined grains and away from coarse grains in China, which might lead to a decreasing intake of Vitamin B, minerals and cereal fibers [33]. As typically refined grains, rice and flour have become the main energy source for the Chinese, accounting for 93% of the total cereal intake [33]. Whole grains have been recommended in many healthy dietary patterns and the dietary guidelines, including the current CDG, as an effective predictor of diabetes, whereas their intake remains low in China. Previous studies have suggested the association of cereal fibre and whole grain intakes with a reduced risk of diabetes with strong evidence, while the non-significant association of refined grains with diabetes were found in many studies [29,34]. For other food groups (salt, oil, meat and egg) with higher contribution rates, only meat (especially red and processed meat) has sufficient evidence on the positive association with the risk of diabetes [5,34]. The role of salts on diabetes risks is still controversial, but most previous studies, though not all, have supported a positive association between a high-salt diet and diabetes risks [35,36]. A recent umbrella review found a non-significant association of diabetes with eggs and an inverse association with olive oil, which is rarely consumed in China [29]. Therefore, for specific components of the HBS, food groups with no significant association of the diabetes risk accounted for the majority, which might lead to no significant association between the HBS and the diabetes risk as well.

As an indicator reflecting the meanings of the LBS and HBS, the DQD evaluates the dietary balance of the Chinese residents by covering the comprehensive food groups, which make it most similar to the aMED, DASH score, AHEI and other typical indexes among the DBI indicators. Our results found an inverse association between the overall diet quality and diabetes risks, which are broadly consistent with previous studies, which reported the beneficial impact of a higher diet quality, assessed by several typical dietary indexes on diabetes prevention. Two systematic reviews indicated that a greater adherence to the Mediterranean diet was associated with 19% to 23% lower risk of diabetes [37,38]. For the HEI, AHEI and DASH scores, Schwingshackl et al. have tracked the latest relative research and reported a significant 18% reduced risk of developing diabetes with higher scores in their second updated systematic reviews [10]. Despite the variety of dietary characteristics among people in different world regions, the antidiabetic effect of healthy diets is also confirmed by two large cohort studies among Chinese populations, which indicated a significant 16% to 29% lower diabetes risks with five dietary pattern scores (aMED, AHEI-2010, DASH scores, PDI and hPDI) [22] and 15% lower diabetes risks with a self-designed dietary index, based on eight commonly consumed food groups across extreme quintiles [23], respectively.

The strengths of our study included the large sample size, the prospective cohort design and a long follow-up period. Furthermore, this study used three-day dietary records to repeat the dietary assessment and the latest DBI, which could better represent the dietary characteristics of Chinese residents to evaluate the participants’ diet quality, and the results are robust in the sensitivity analyses.

Nevertheless, our study has several limitations. First, as in all observational studies, measurement errors are inevitable. Repeat measures would overcome some of the errors, and the use of cumulative averages of diet would provide a better measure of the participants’ long-term diet quality. Second, the diabetes incidence might be underestimated for participants with only self-reports from the questionnaires, which might have resulted in the misclassification of the outcome. Meanwhile, this study didn’t distinguish between type 1 and type 2 diabetes, even though more than 90% of diabetics are diagnosed with type 2 diabetes [39]. Finally, although we carefully filled in and adjusted for the various potential confounders, which was supported by the results of the second sensitive analysis, the residual and unmeasured confounding remains possible.

In summary, we observed a significant inverse association between the diet quality assessed by the DBI-16 and the diabetes risks. Our findings provide evidence for the positive role of healthy diets in the diabetes prevention in Asia, especially in China, which is facing large dietary transitions, as well as the rapid growth of diabetes burdens.

## Figures and Tables

**Table 1:** Contribution rates of the DBI-16 sub-components on the LBS, HBS and DQD.

LBS	%	HBS	%	DQD	%
Dairy	21.37	Cereal	48.48	Cereal	19.40
Diet variety	20.74	Salt	18.97	Dairy	13.00
Fruit	19.13	Oil	16.37	Diet variety	12.61
Fish	10.18	Meat	11.01	Fruit	11.64
Soybean	9.97	Egg	3.92	Salt	7.44
Egg	7.55	Alcohol	0.82	Meat	6.54
Vegetable	6.74	Sugar	0.42	Oil	6.41
Meat	3.65			Fish	6.19
Cereal	0.66			Egg	6.13
				Soybean	6.06
				Vegetable	4.10
				Alcohol	0.32
				Sugar	0.17

**Table 2:** Baseline characteristics of the participants on the different LBS, HBS, DQD levels (*N* = 9394).

Characteristics	LBS Levels				*p* Value	HBS levels				*p* Value	DQD Levels			*p* Value
	L1	L2	L3	L4		L1	L2	L3	L4		L1&2	L3	L4	
*N*	384	3847	4858	305		591	6133	2565	105		1718	6508	1168	
Score range	0–12	13–24	25–36	>36		0–9	10–18	19–27	>27		0–34	35–50	>50	
Age(years)	53.0 [39.8; 60.0]	48.0 [38.0; 57.0]	48.0 [39.0; 58.0]	51.0 [41.0; 62.0]	<0.001	49.0 [38.0; 59.0]	48.0 [39.0; 57.0]	49.0 [39.0; 58.0]	47.0 [38.0; 55.0]	0.070	49.0 [38.0; 58.0]	48.0 [39.0;57.0]	51.0 [41.0;59.0]	<0.001
Male (%)	38.8	45.6	47.1	47.2	0.012	40.1	44.7	50.4	62.9	<0.001	39.2	47.2	50.4	<0.001
Urban site (%)	88.5	58.5	26.2	22.0	<0.001	63.1	38.0	45.7	48.6	<0.001	71.1	37.5	22.8	<0.001
Education level (%)					<0.001					<0.001				<0.001
Primary school	8.6	24.4	47.5	65.2		19.1	38.7	37.3	28.6		14.1	39.7	55.7	
Middle school	23.7	30.7	32.6	22.6		27.2	31.2	31.9	33.3		27.8	32.3	29.8	
High school or above	67.7	44.9	19.9	12.1		53.6	30.1	30.8	38.1		58.1	28.0	14.5	
Occupation (%) #					<0.001					<0.001 *				<0.001
Farmer or worker	13.3	33.8	69.5	84.7		30.8	57.0	54.7	47.0		22.7	57.1	80.3	
Service staff	15.7	22.4	13.4	8.1		17.4	16.2	17.6	20.5		18.5	17.8	8.3	
Managers or technicians	62.9	37.2	11.8	6.4		43.7	21.6	21.3	27.7		53.1	18.8	8.5	
Other	8.1	6.6	5.3	0.8		8.1	5.3	6.4	4.8		5.7	6.3	3.0	
Smoking (%)	22.1	29.6	33.5	34.4	<0.001	25.5	30.5	34.8	41.0	<0.001	23.9	32.3	38.1	<0.001
Alcohol drinking(%)	34.9	35.8	33.5	29.2	0.034	30.3	32.8	38.1	56.2	<0.001	33.8	34.2	36.0	0.408
BMI(kg/m^2^)	23.9 [21.9; 26.1]	23.7 [21.4; 25.9]	23.0 [21.0; 25.2]	23.0 [20.5; 24.8]	<0.001	23.4 [21.2; 25.6]	23.2 [21.1; 25.4]	23.7 [21.5; 25.9]	23.4 [21.4; 26.0]	<0.001	23.6 [21.5; 25.9]	23.4 [21.2;25.5]	23.0 [21.0;25.2]	<0.001
Physical activity					<0.001									<0.001
Inadequate	33.1	34.7	31.9	33.1		36.0	33.7	31.8	18.1		36.0	32.8	30.7	
Low	8.3	4.5	2.6	0.7		7.3	3.0	3.9	8.6		6.2	2.9	3.2	
Moderate or high	58.6	60.8	65.5	66.2		56.7	63.3	64.3	73.3		57.8	64.2	66.2	
Dietary intake														
Total energy (kcal/day)	1774 [1481; 2144]	1970 [1638; 2327]	2050 [1692; 2418]	1789 [1395; 2211]	<0.001	1973 [1692; 2314]	2055 [1749; 2392]	1842 [1399; 2312]	1425 [1145; 1673]	<0.001	1962 [1674; 2274]	2027 [1675;2402]	1877 [1432;2325]	<0.001
Fat(g/day)	78.2 [66.9; 91.4]	77.4 [66.6; 90.6]	67.4 [53.8; 80.7]	55.8 [38.9; 71.2]	<0.001	83.2 [71.6; 96.4]	70.7 [57.0; 84.5]	72.7 [60.0; 85.8]	80.9 [70.9; 92.5]	<0.001	80.1 [68.3; 93.3]	71.7 [58.5;84.9]	61.7 [47.5;77.4]	<0.001
Carbohydrate (g/day)	243 [213; 270]	257 [227; 283]	290 [257; 322]	323 [286; 360]	<0.001	240 [212; 267]	279 [244; 312]	272 [239; 302]	238 [210; 265]	<0.001	246 [218; 273]	277 [244;308]	307 [266;340]	<0.001
Protein(g/day)	82.2 [73.8; 92.4]	69.3 [63.0; 77.4]	59.9 [54.6; 66.3]	53.6 [48.5; 59.2]	0.000	71.7 [61.5; 84.0]	63.2 [56.8; 70.9]	64.9 [57.7; 73.3]	70.9 [63.1; 78.7]	<0.001	74.0 [66.3; 84.3]	63.3 [57.3;70.3]	56.8 [50.5;63.2]	0.000
Hypertension (%)	22.7	11.4	9.2	7.5	<0.001	14.4	9.8	11.7	10.5	0.001	15.0	9.6	9.4	<0.001

Note: Data are median (IQR) for the continuous variables. Information on the non-dietary factors was collected at the baseline, and the dietary data were estimated as the energy-adjusted cumulative average intake from the baseline and follow-up periods. We used Kruskal–Wallis tests for the continuous variables and the χ^2^ tests for the categorical variables (except * which used the Kruskal–Wallis test). # had missing data.

**Table 3:** The relative risk of developed diabetes, based on the levels of the LBS, HBS and DQD (*N* = 9394).

Levels of the DBI	Score Range	N	Cases/Person-Years	Model 1	Model 2	Model 3
				HR (95% CI)	HR (95% CI)	HR (95% CI)
LBS						
L1	0–12	384	12/1614	Ref	Ref	Ref
L2	13–24	3847	250/23,585	2.35 [1.31, 4.21]	2.27 [1.27, 4.08]	2.43 [1.36, 4.37]
L3	25–36	4858	365/35,228	2.67 [1.48, 4.84]	2.58 [1.43, 4.68]	3.05 [1.69, 5.53]
L4	>36	305	30/1552	3.97 [1.99, 7.92]	3.86 [1.94, 7.70]	4.90 [2.46, 9.78]
HBS						
L1	0–9	591	33/2428	Ref	Ref	Ref
L2	10–18	6133	406/44,459	1.02 [0.71, 1.46]	0.99 [0.69, 1.43]	1.06 [0.74, 1.51]
L3	19–27	2565	213/14,700	1.35 [0.93, 1.96]	1.33 [0.92, 1.93]	1.30 [0.90, 1.88]
L4	>27	105	5/391	0.93 [0.36, 2.38]	0.94 [0.37, 2.42]	0.99 [0.39, 2.55]
DQD						
L1&2	0–34	1718	95/8969	Ref	Ref	Ref
L3	35–50	6508	445/45,987	1.23 [0.97, 1.56]	1.21 [0.96, 1.54]	1.28 [1.01, 1.61]
L4	>50	1168	117/7023	1.88 [1.40, 2.53]	1.88 [1.39, 2.52]	2.10 [1.57, 2.82]

Note: Model 1: adjusted for age, sex, education level, and urban or rural residence at baseline. Model 2: model 1 + physical activity, smoking and alcoholic drinking. Model 3: model 2 + body mass index (BMI), and hypertension.

## Data Availability

The dataset in the present study was open-access and can be freely obtained from the CHNS website: https://www.cpc.unc.edu/projects/china/data/datasets/data_downloads/longitudinal (accessed on 12 October 2022).

## Supplementary Materials

The following supporting information can be downloaded at: https://www.mdpi.com/article/10.3390/nu14224841/s1, Figure S1: Flow chart of the study; Table S1: First sensitivity analysis of the relative risk of developed diabetes, based on the levels of the LBS, HBS and DQD (N = 12107); Figure S2: First sensitivity analysis of the relation between the (A) LBS, (B) HBS, and (C) DQD with the risks of diabetes; Table S2: The relative risk of developed diabetes, based on the levels of the LBS, HBS and DQD (N = 6344); Figure S3: Second sensitivity analysis of the relation between the (A) LBS, (B) HBS, and (C) DQD with the risks of diabetes.
