# Supplementary material for: Long-Term Diet Quality and Risk of Diabetes in a National Survey of Chinese Adults
Source: Nutrients. 2022 Nov 16;14(22):4841. doi: 10.3390/nu14224841 (PMC9695385; doi:10.3390/nu14224841)
Supplement: Supplementary file 1 [file nutrients-14-04841-s001.zip › nutrients-supplementary-Proofreading.pdf]

Supplementary Materials

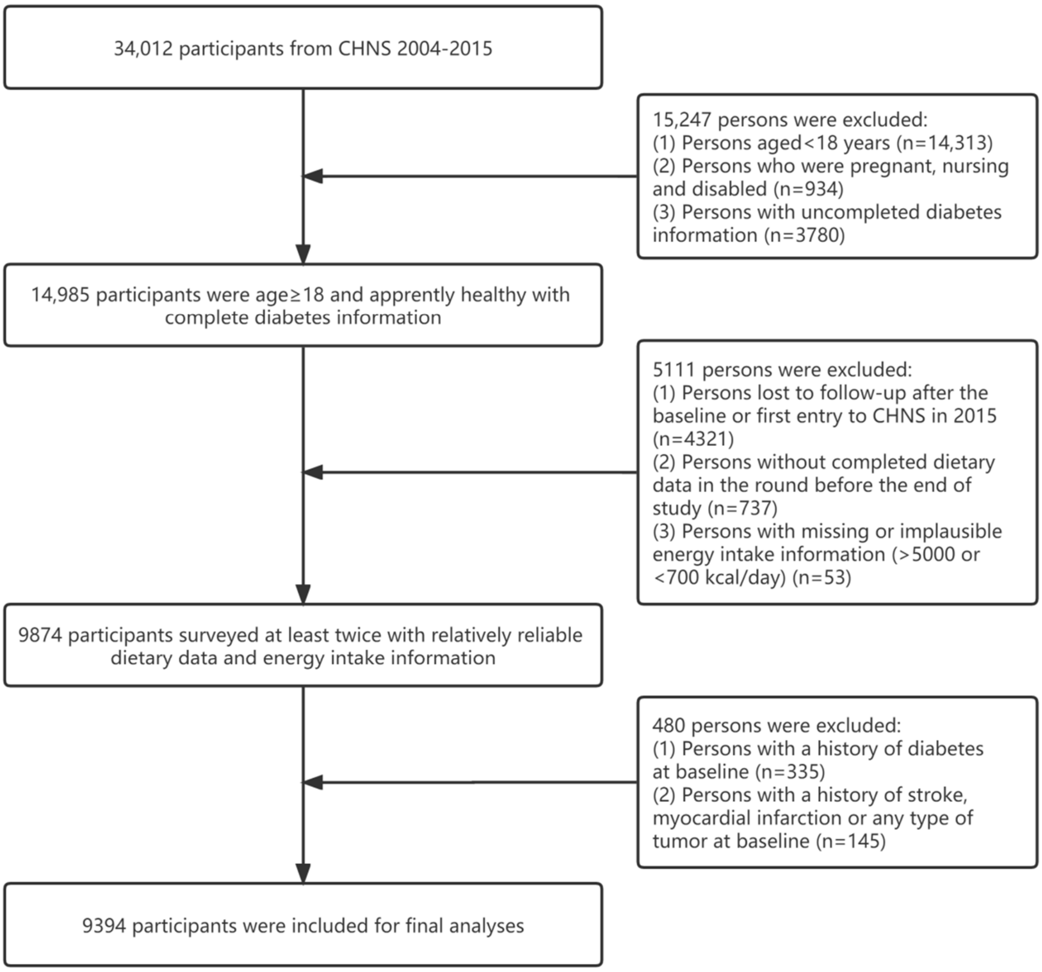

Figure S1. Flow chart of the study.

Table S1. First sensitivity analysis of the relative risk of developed diabetes based on the levels of LBS, HBS and DQD (N=12,107).

Levels of DBI	Score range	N	Cases/person-years	Model 3
				HR(95% CI)
LBS				
L1	0-12	445	14/1840	Ref
L2	13-24	4631	271/27390	2.12 [1.23, 3.64]
L3	25-36	6504	440/43524	2.69 [1.55, 4.66]
L4	>36	527	44/2311	4.65 [2.50, 8.67]
HBS				
L1	0-9	733	37/2904	Ref
L2	10-18	7886	486/53633	1.04 [0.74, 1.46]
L3	19-27	3358	240/18057	1.22 [0.86, 1.73]
L4	>27	130	6/470	1.02 [0.43, 2.43]
DQD				
L1&2	0-34	2020	103/10316	Ref
L3	35-50	8337	518/55263	1.25 [1.00, 1.56]
L4	>50	1750	148/9485	1.99 [1.51, 2.61]

Note: Scores of three DBI indicators was calculated using the dietary data from baseline and divided into 4 levels according to the same methods of Table 3 in the article. HRs and 95% CIs were adjusted for the variables listed for model 3 of Table 3.

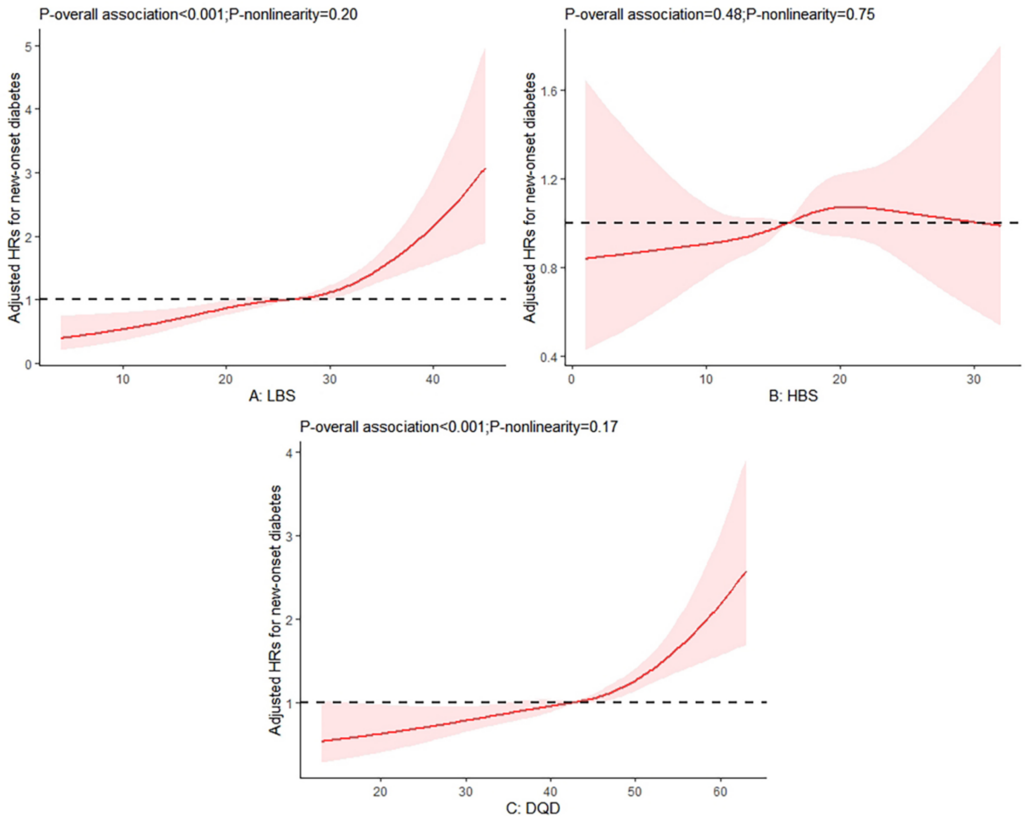

Figure S2. First sensitivity analysis of the relation of (A) LBS, (B) HBS, and (C) DQD with the risks of diabetes.

Note: Adjusted for age, sex, education level, urban or rural residence, physical activity, smoking, alcoholic drinking, body mass index (BMI), as well as hypertension.

Table S2. Second sensitivity analysis of the relative risk of developed diabetes based on the levels of LBS, HBS and DQD (N=6344).

Levels of DBI	Score range	N	Cases/person-years	Model 3
				**HR(95% CI)**
LBS				
L1	0-12	337	12/1424	Ref
L2	13-24	2952	160/17614	1.94 [1.07, 3.50]
L3	25-36	2924	204/20631	2.76 [1.50, 5.07]
L4	>36	131	11/664	4.31 [1.85, 10.04]
HBS				
L1	0-9	480	26/1976	Ref
L2	10-18	4044	226/28389	0.96 [0.64, 1.45]
L3	19-27	1740	133/9669	1.28 [0.83, 1.95]
L4	>27	80	2/297	0.55 [0.13, 2.31]
DQD				
L1&2	0-34	1439	68/7476	Ref
L3	35-50	4269	257/29144	1.38 [1.04, 1.83]
L4	>50	636	62/3712	2.53 [1.74, 3.67]

Note: Scores of three DBI indicators was calculated using the dietary data from baseline and divided into 4 levels according to the same methods of Table 3 in the article. HRs and 95% CIs were adjusted for the variables listed for model 3 of Table 3.

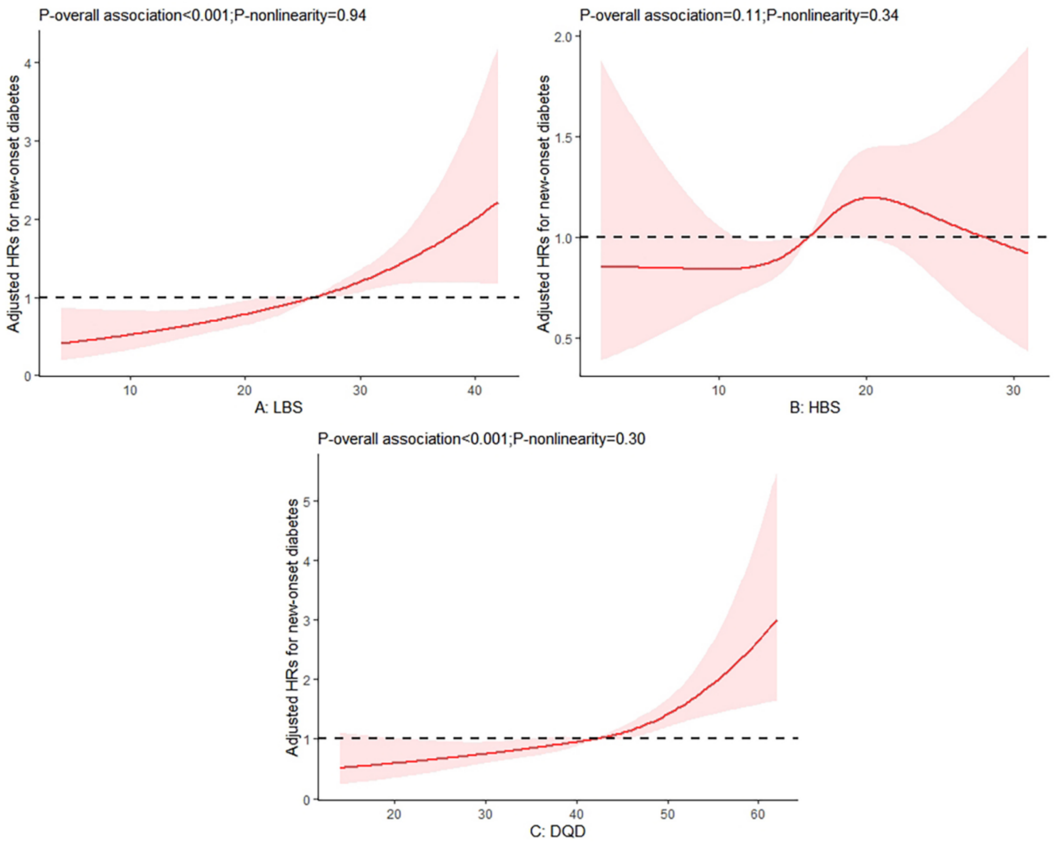

Figure S3. Second sensitivity analysis of the relation of (A) LBS, (B) HBS, and (C) DQD with the risks of diabetes.

Note: Adjusted for age, sex, education level, urban or rural residence, physical activity, smoking, alcoholic drinking, body mass index (BMI), as well as hypertension.
